# Tunneling in Systems of Coupled Dopant-Atoms in Silicon Nano-devices

**DOI:** 10.1186/s11671-015-1076-z

**Published:** 2015-09-24

**Authors:** Daniel Moraru, Arup Samanta, Krzysztof Tyszka, Le The Anh, Manoharan Muruganathan, Takeshi Mizuno, Ryszard Jablonski, Hiroshi Mizuta, Michiharu Tabe

**Affiliations:** Department of Electronics and Materials Science, Faculty of Engineering, Shizuoka University, Shizuoka, Japan; Research Institute of Electronics, Shizuoka University, Shizuoka, Japan; Institute of Metrology and Biomedical Engineering, Warsaw University of Technology, Sw. A Boboli 8, Warsaw, Poland; School of Materials Science, Japan Advanced Institute of Science and Technology (JAIST), Kanazawa, Japan; Faculty of Physical Sciences and Engineering, University of Southampton, Southampton, UK

**Keywords:** Single-dopant, Dopant-cluster, SOI-FET, Single-electron tunneling, KPFM

## Abstract

Following the rapid development of the electronics industry and technology, it is expected that future electronic devices will operate based on functional units at the level of electrically active molecules or even atoms. One pathway to observe and characterize such fundamental operation is to focus on identifying isolated or coupled dopants in nanoscale silicon transistors, the building blocks of present electronics. Here, we review some of the recent progress in the research along this direction, with a focus on devices fabricated with simple and CMOS-compatible-processing technology. We present results from a scanning probe method (Kelvin probe force microscopy) which show direct observation of dopant-induced potential modulations. We also discuss tunneling transport behavior based on the analysis of low-temperature *I*-*V* characteristics for devices representative for different regimes of doping concentration, i.e., different inter-dopant coupling strengths. This overview outlines the present status of the field, opening also directions toward practical implementation of dopant-atom devices.

## Review

### Introduction

Within the past few decades, silicon metal-oxide-semiconductor field-effect transistors (MOSFETs) have undergone a tremendous miniaturization process [1, 2] which brings us within the era of nanoelectronics implemented in practical electronic and photonic devices. Commercially available transistors have now minimum features on the order of only a few tens of nanometers, a feat possible thanks to the great progress of nanotechnology but also to our increasing understanding of device operation at these extreme scales. Not only silicon [3, 4], but also two-dimensional materials [5–8], continue to exhibit interesting and novel functionalities when designed as MOSFETs. It is expected that further control in nanoscale will be achieved in the near future based on the steady improvement of knowledge and technology. However, it is also expected that the conventional operation mechanism of transistors—mainly based on drift-diffusion current flow—will be replaced by more fundamental physics [9, 10], such as quantum tunneling, which becomes a dominant phenomenon in nanoscale and ultimately at molecular and atomic scales as well. As transistors working based on conventional operation approach the end of their history, it becomes clear that alternatives must be developed and investigated for future generations of electronics. As a bridge between regular silicon-based nanoelectronics and future electronics at real molecular and atomic scale, a number of groups focus on the development of dopant-atom silicon nano-transistors and related devices [11–16]. Several state-of-the-art techniques have also been used to demonstrate proof-of-concept devices, either by using single-ion implantation (SII) technique [17] or using a scanning tunneling microscope (STM) tip atomic manipulation of Si surfaces [18]. These results offer important insights into the fundamental levels of controllability at atomic scale, but they remain significantly complex and incompatible with CMOS processing technology.

In this Nano Review, we briefly outline the key aspects that must be addressed in order to clarify and improve the tunneling operation of dopant-atom devices. We will mainly focus on techniques that allow fabrication of dopant-atom devices with relative ease. For our devices, we use a thermal-diffusion doping technique that allows only a statistical control of the average number of dopants in the transistor channel. We also introduce selective-doping techniques that could allow, after further optimization, additional control of design parameters of silicon nano-transistors in different regimes of inter-dopant interaction strength [19].

We first show how individual or coupled dopants modulate the potential landscape in the channel of silicon nano-devices. For that purpose, we describe results obtained using a specially designed Kelvin probe force microscopy (KPFM) technique [20]. We then show how dopant atoms or clusters of strongly-coupled dopants control the quantum-tunneling conductance in nanoscale transistors doped in different regimes of doping concentration. In that sense, we analyze low-temperature electrical characteristics that exhibit signatures of single-electron tunneling (SET) mediated by dopant-induced quantum dots (QDs) [19]. Depending on the internal structure of the QD, i.e., number and interaction strength among dopants forming the QD, the current peaks exhibit distinct properties, illustrating electron transport via either atomic- or molecular-like structures.

### Observation of Dopant-Induced Quantum Dots

The devices that we investigate are silicon-on-insulator field-effect transistors (SOI-FETs) with the channel usually doped with phosphorus (P) donors by thermal-diffusion doping. In silicon nano-transistors doped by such conventional doping technique, number and position of dopants in the channel cannot be precisely controlled. In order to understand the impact of dopants and of their distribution on the electrical properties of nano-transistors, it is essential to observe directly the modulation of the electronic potential induced by the dopants. For this purpose, we use a KPFM technique, specially designed to meet the requirements for measurements of dopants in devices under regular operation.

**Fig. 1:**
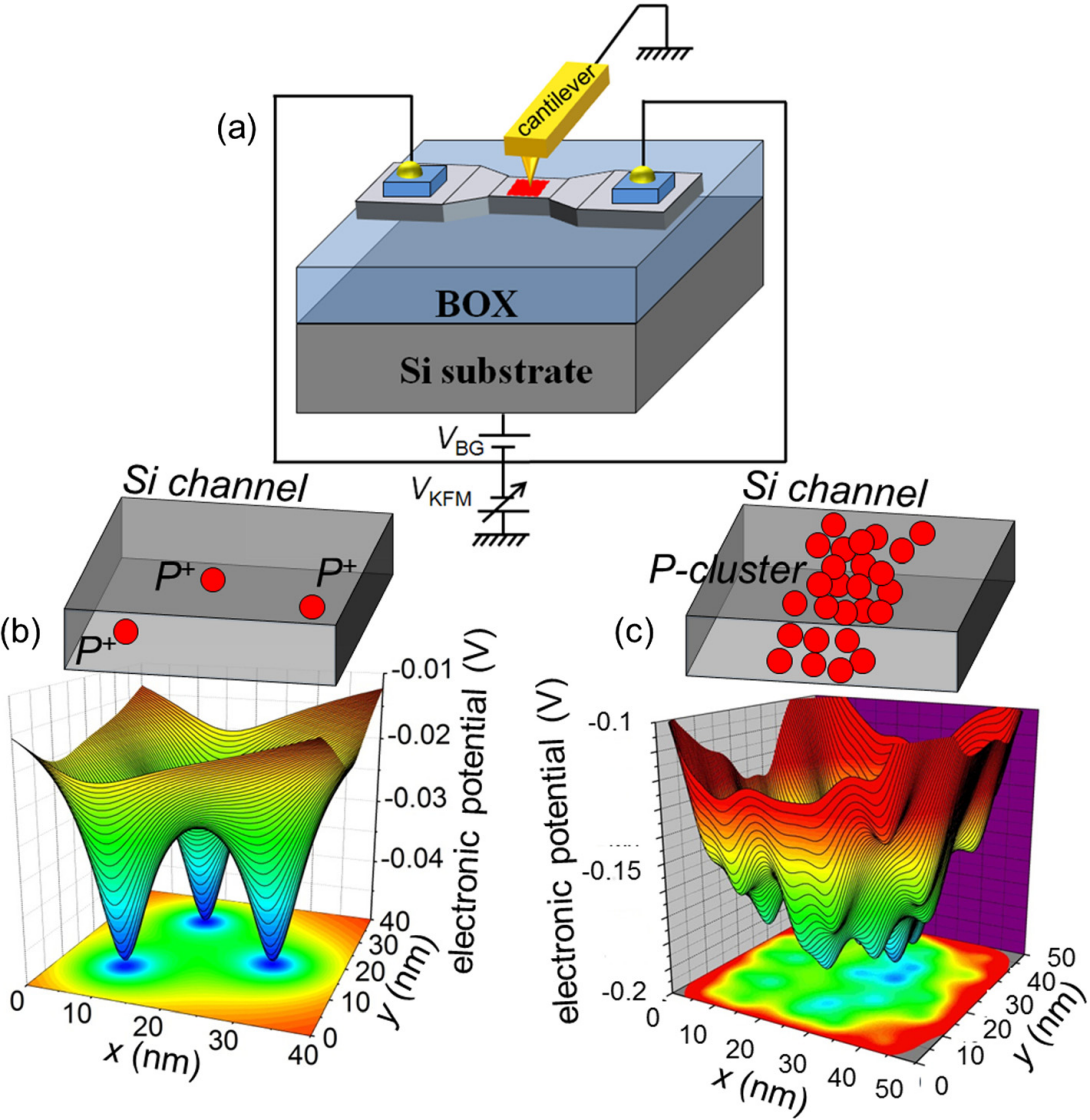
Kelvin probe force microscopy of donor atoms. **a** KPFM measurement setup, showing a cantilever approached near the surface of a SOI-FET channel with the device under regular operation conditions. **b** A possible potential landscape induced by several isolated, ionized P-donors. **c** A possible potential landscape induced by a larger number of P-donors forming multiple-donor “clusters” (containing several donors located at distances smaller than 2 × *r*_B_ from each other)

KPFM is a suitable technique for such measurements because its detection principle is based on sensing the electrostatic force between a metallic tip (cantilever) and any point charges located in the sample [21], as schematically illustrated in Fig. 1a. However, there are several conditions that must be met in order to be able to observe dopants in a transistor nano-channel [20, 22]. First, the dopants should be ionized, i.e., for the case of a phosphorus-doped device, electrons should be depleted from the channel. For that purpose, the SOI-FETs have the source and drain leads connected to the ground, while negative voltage is applied to the back gate (substrate Si) in order to remove the free electrons from the channel to the leads. Thus, ionized P-donors are left behind as fixed positive point charges. Second, in order to further remove unwanted screening effects due to thermally-activated carriers, the KPFM measurements can be carried out even at low temperatures in our system. Third, the Si layer should be passivated (to avoid dangling bonds and other defect states that could affect the measurement). Such passivation is done by thermal oxidation in our fabrication processes. However, the SiO_2_ layer should be thin enough (usually, ~1 nm) to allow for the cantilever to approach the Si surface at a distance at which the electrostatic force induced by the dopants located in the channel can still be detected with sufficiently high resolution.

These requirements are all taken into consideration when designing our devices and the KPFM measurement system, allowing thus the observation of dopants present in the device channel with the free carriers removed. Depending on the doping concentration and channel design, it is possible to observe either isolated, individual P-donors (as schematically shown in Fig. 1b) or clusters of P-donors (as schematically shown in Fig. 1c).

#### Individual, Isolated Dopants in Low-Concentration Regime

In several of our previous publications, we reported the measurements of potential landscapes for devices with the channel doped with P-donors at an intermediate doping concentration level (*N*_D_ ≈ 1 × 10^18^ cm^−3^) [20, 22–24]. For this concentration regime, average distance between neighboring P-donors is ~10 nm (> > 2 × *r*_B_ ≈ 5 nm, with *r*_B_ being the Bohr radius for P in Si). Thus, if P-donors are distributed according to a Poisson distribution, it is likely to find them reasonably isolated from each other, each locally modulating the potential of the channel. On the other hand, the potential modulation induced by such isolated P-donors is relatively low (only a few tens of meV).

**Fig. 2:**
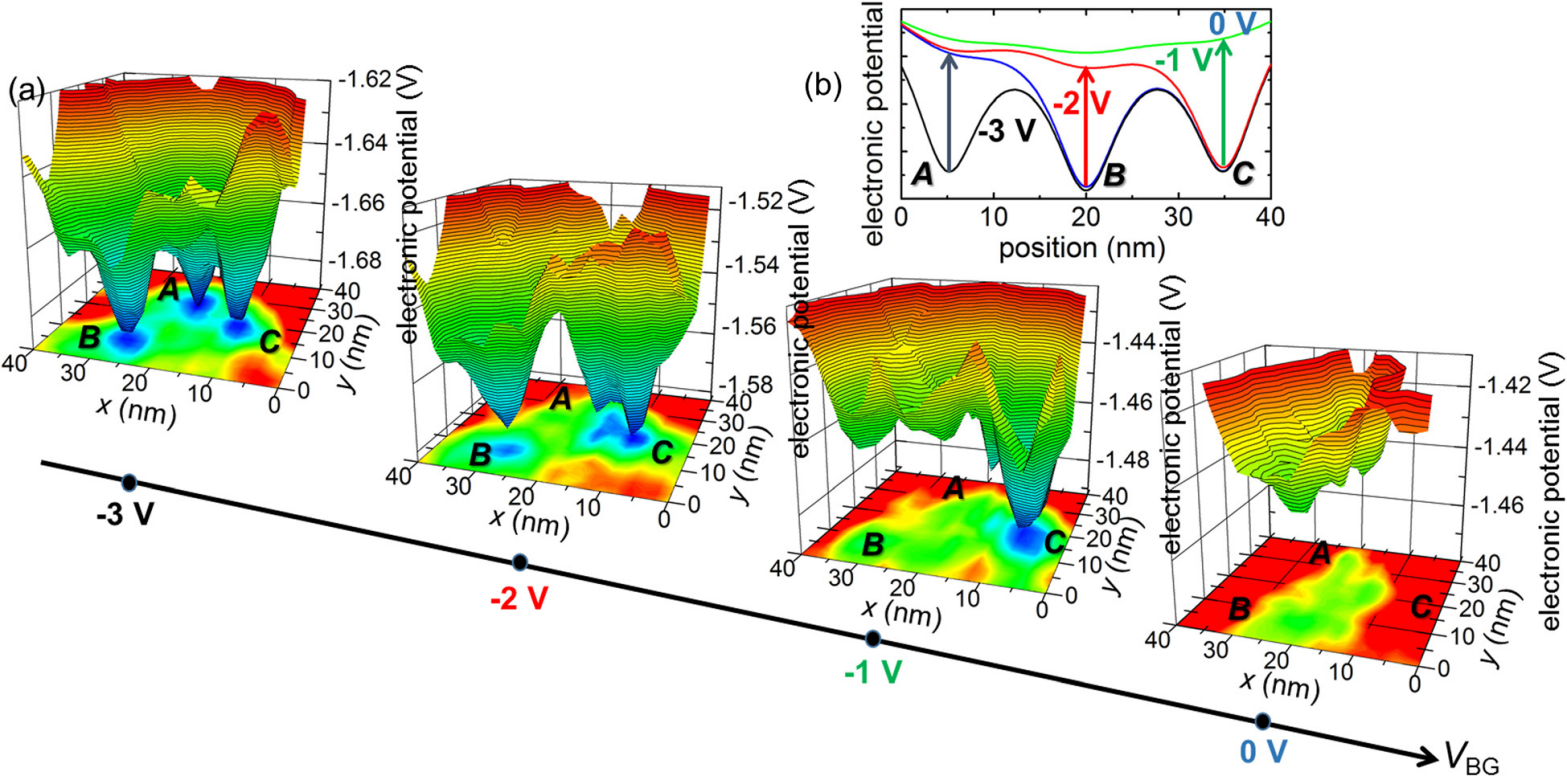
Electron injection in individual donor atoms observed by KPFM. **a** Sequence of electronic potential landscapes measured at low temperature (*T* = 13 K) on the channel of an SOI-FET doped with P-donors (*N*_D_ ≈ 1 × 10^18^ cm^−3^) as a function of applied *V*_BG_ (−3 ~ 0 V). **b** A simple illustration of one-by-one neutralization of individual P-donors at different *V*_BG_s [23]

Figure 2a shows a sequence of KPFM measurements at low temperature (*T* = 13 K) on an area within the channel of an SOI-FET for which an appropriate arrangement of P-donors was actually identified [23]. In particular, isolated P-donor-induced potential wells can be clearly observed for most negative *V*_BG_ (−3 V). Interestingly, if *V*_BG_ is increased in the positive direction, the potential wells vanish one by one at successive values of *V*_BG_, with no significant features remaining at *V*_BG_ = 0 V. As illustrated in the schematic model (Fig. 2b), this can be interpreted as successive captures of single electrons in individual P-donors. In such a simplified interpretation, this picture is consistent with the concept that individual P-donors work as distinct QDs in single-electron tunneling transport. This concept is at the core of the operation principle for single-dopant transistors, and our direct observation provides thus a straightforward visualization of such fundamental events.

We have also shown that, by increasing the temperature, free-electron screening becomes more dominant than the capture of electrons into different P-donors [23]. This explains, based on more direct observations, why dopant-atom devices in which shallow P-donors are the active units in transport cannot work based on tunneling mechanism at elevated temperatures. An alternative approach to achieve elevated-temperature tunneling operation based on individual P-donors will be discussed in a subsequent section.

#### Coupled Dopants in High-Concentration Regime

In order to implement more complex functionalities, as well as to design more robust dopant-induced QDs, the QDs can be formed not by individual P-donors but by a number of P-donors placed closely to each other (within 2 × *r*_B_). In order to promote this regime, it is first of all required to increase the doping concentration (*N*_D_), ideally to values above the metal-insulator transition (MIT) concentration (for our design, typical concentration used is *N*_D_ ≈ 1 × 10^19^ cm^−3^) [19].

Under such conditions, due to the strong interaction between neighboring P-donors, “clusters” of a larger number of donors will contribute to the formation of the QDs. It is, however, also required to isolate the highly doped QDs from source and drain leads in order to ensure the possibility of depleting the (quasi-metallic) channel. For that purpose, we use a selective-doping technique [19], in which a SiO_2_ doping mask is patterned by an electron beam lithography technique to preserve two fine non-doped regions acting as barriers on the sides of a highly doped slit. This way, the channel is doped only locally and, at the same time, self-aligned with source and drain leads, all regions having a doping concentration (*N*_D_) on the order of 1 × 10^19^ cm^−3^.

**Fig. 3:**
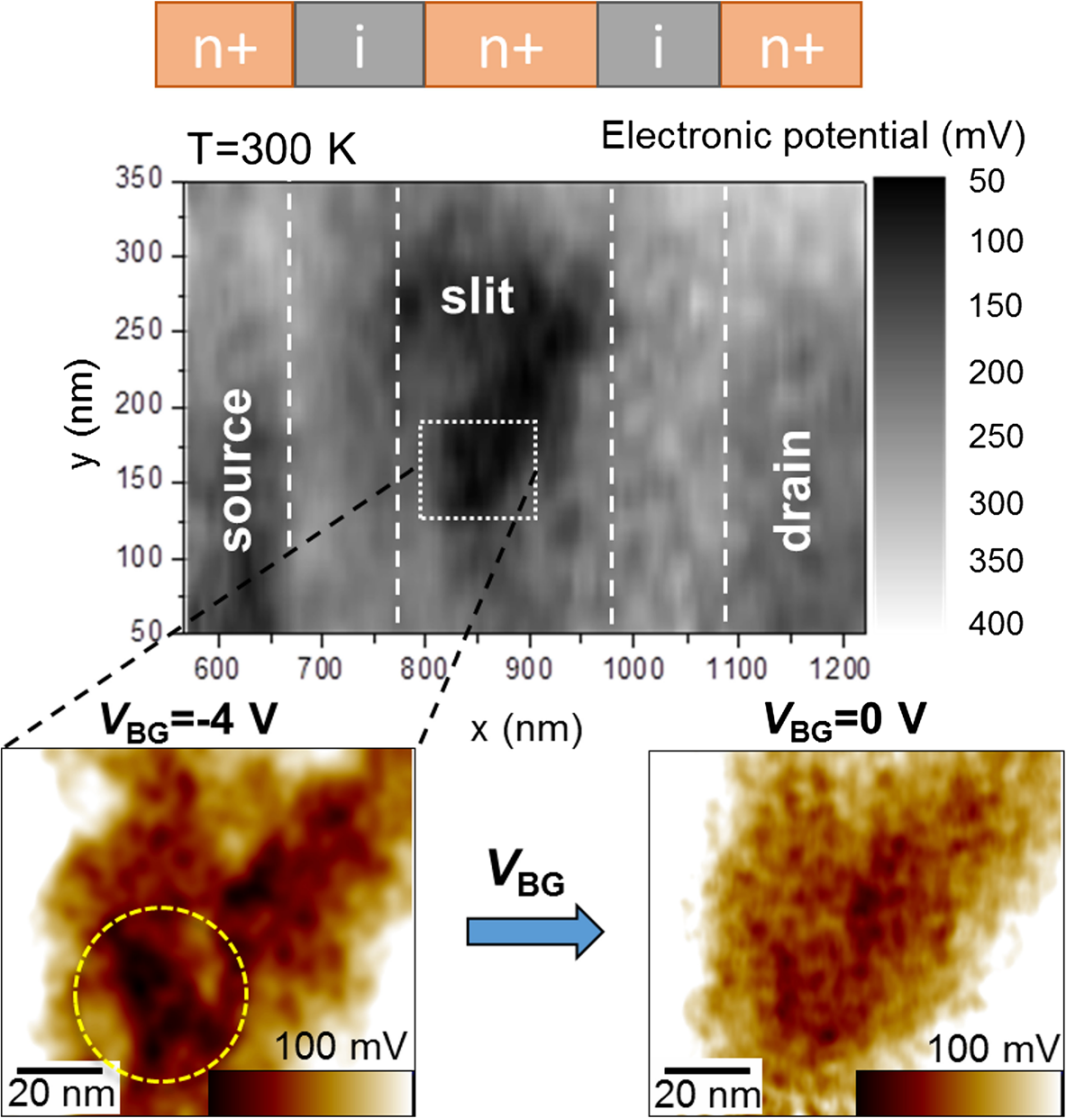
Donor-cluster-induced potential modulations observed by KPFM. KPFM measurement of the electronic potential map for a selectively doped high-*N*_D_ SOI-FET channel (with doping profile indicated on the *top panel*). Doping concentration for these devices is estimated to be *N*_D_ ≈ 1 × 10^19^ cm^−3^. Inside the P-doped slit, dark-contrast, deep-potential regions can be seen for *V*_BG_ = −4 V (marked by a *dashed circle* in the *left panel*) but are smeared out for *V*_BG_ = 0 V (*right panel*)

For this type of devices, as shown in Fig. 3, we can observe the potential landscape in correlation with regions defined as source, drain, and P-doped slit, identified based on correlation with the doping profile (indicated in the upper panel). The KPFM measurements shown here were taken at room temperature (*T* = 300 K). It can be seen from Fig. 3 that the heavily-doped (*N*_D_ ≈ 1 × 10^19^ cm^−3^) region (slit) has a lower electronic potential than the nominally non-doped regions. Furthermore, as marked in the lower zoom-in panel for negative *V*_BG_, it is possible to identify fine modulations of the potential inside the heavily-doped slit. These features can be ascribed to “clusters” of several P-donors grouped together inside the selectively-doped region, according to our detailed analysis correlated with dopant-induced potential simulations [25]. It is found that such clusters (containing even more than 10 P-donors) can work as dominant QDs in the tunneling transport characteristics, with a significant effect of the selective-doping technique in controlling the QD position within the channel [26]. As *V*_BG_ is made more positive, free carriers start to screen the potential and the contrast becomes significantly reduced. Thus, KPFM could also provide information about the distribution and properties of dopant-induced QDs in this more complex regime in which a number of P-donors strongly interact with each other. This regime is of particular interest for applications that aim at utilizing the molecular behavior of such multiple P-donor “clusters” in Si nanostructures.

### Single-Electron Tunneling via Dopant-Induced QDs

**Fig. 4:**
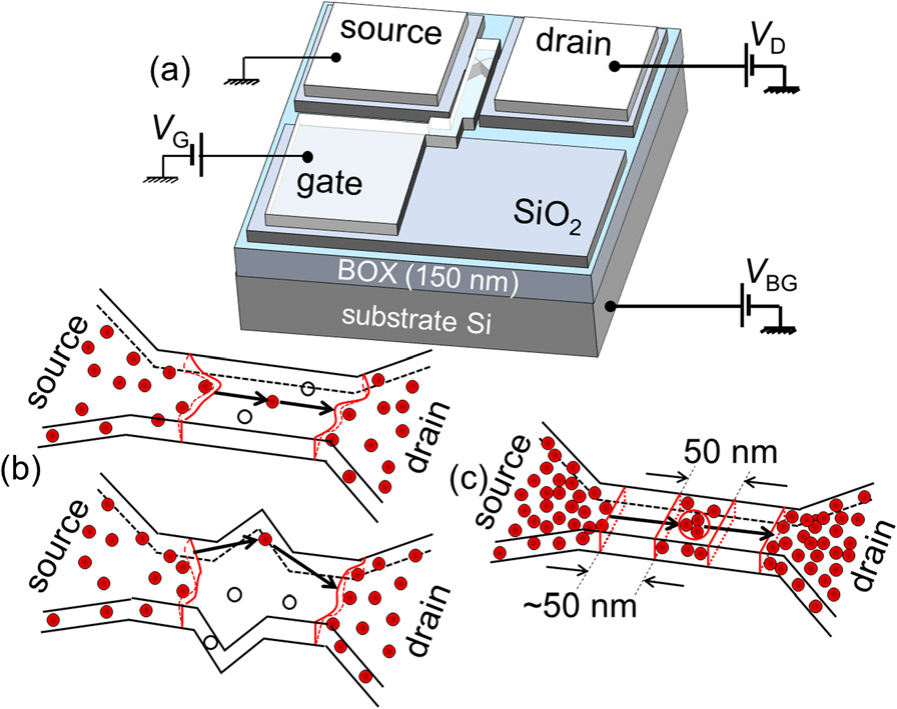
Nanoscale-channel transistors with various channel designs. **a** SOI-FETs with a top gate and bias circuit for *I*_D_-*V*_G_ measurements. Different devices have been fabricated, with different doping concentrations and profiles across the channel: **b** low concentration (*N*_D_ ≈ 1 × 10^18^ cm^−3^)—single-electron tunneling occurs via individual P-donors (nanowire and stub-shaped channels are illustrated); **c** high concentration (*N*_D_ ≈ 1 × 10^19^ cm^−3^)—single-electron tunneling occurs via “clusters” of P-donors

In order to reveal the electrical properties of various systems of dopants, either isolated or “clustered,” it is common to measure source-drain current (*I*_D_) versus gate voltage (*V*_G_) characteristics, in particular at low temperatures (< <100 K) for doped-channel nanoscale SOI-FETs, with a bias setup as shown in Fig. 4a. At low temperature, as discussed earlier, thermal activation of carriers can be minimized and conductance occurs dominantly by tunneling via dopant-states.

Typically, a small source-drain bias (*V*_DS_ = 5 mV) is applied. The *I*_D_-*V*_G_ characteristics exhibit, under such conditions, current peaks or current modulations, especially near the onset of the conduction. Such current peaks can be ascribed to Coulomb blockade transport through QDs existing in the device channel. The origin of the QDs is, at its turn, related to the P-donors purposely doped into the channel. Depending on the doping concentration regime and device design, different behaviors can be observed, allowing observation of single-electron tunneling via individual P-donors (as schematically illustrated in Fig. 4b) or multiple-donor clusters (as shown in Fig. 4c).

#### Tunneling Transport via Single Dopant-Atoms

In the lower-concentration regime (*N*_D_ ≈ 1 × 10^18^ cm^−3^), it is expected that individual P-donors work as distinct QDs. As explained earlier, this assumption is most reasonable because, for this doping concentration regime, the average distance between neighboring P-donors is significantly larger that 2 × *r*_B_. Under such conditions, clusters of several donors are statistically unlikely to be formed. For long channels, the only way to realize tunneling conduction between source and drain would be by tunneling via an array of capacitively coupled P-donors. This condition can be useful for the design of a number of applications, such as dopant-based single-electron turnstiles [27, 28] or single-electron pumps [29, 30], but the behavior of such systems is quite complex. However, if the channel is short enough, it may be possible to identify a single P-donor as the dominant QD which completely controls tunneling transport between source and drain. Such a situation is schematically shown for a nanowire-channel SOI-FET in Fig. 4b. However, as long as the P-donors responsible for such transport are expected to be regular, shallow donors, tunneling transport is also expected to vanish at intermediate temperatures due to the significant contribution from thermally-activated carriers [31].

In order to enhance the tunneling-operation temperature, it is necessary to increase, first of all, the tunnel barriers. One way to do that is to take advantage of the dielectric confinement effect. It has been reported in a number of papers that dielectric confinement effect (when donors are embedded in nanostructures closely surrounded by an insulator) leads to a significant increase of the P-donors’ ionization energy [32–34]. In other words, the P-donors’ ground state becomes significantly deeper below the conduction band edge. Thus, for *V*_G_ values corresponding to tunneling via the P-donor’s ground state, thermally activated conduction should be, in principle, suppressed even up to higher temperatures.

**Fig. 5:**
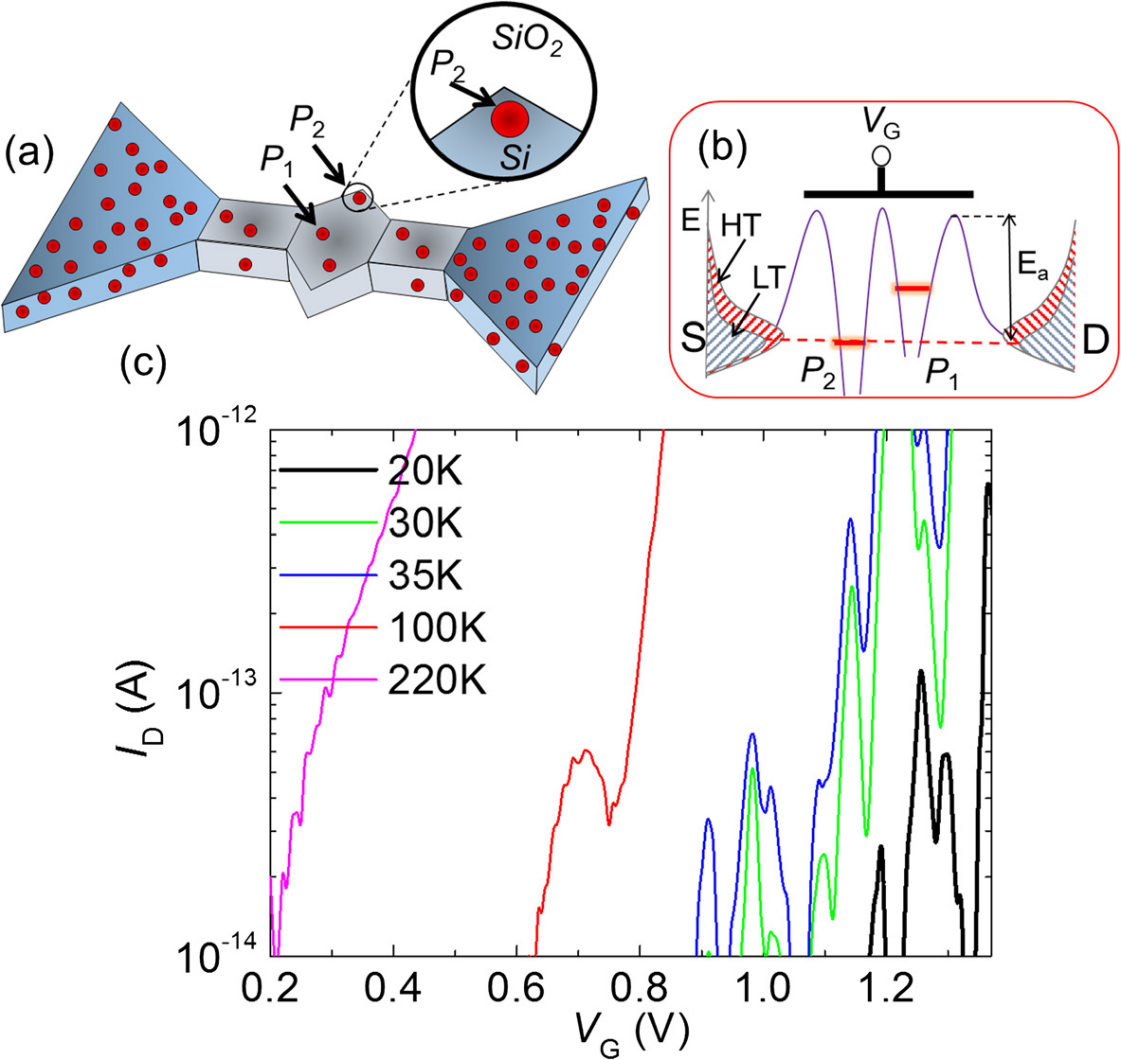
Single-electron tunneling operation of single-dopant transistors at elevated temperatures. **a** Stub-channel SOI-FET (doped with a doping concentration *N*_D_ ≈ 1 × 10^18^ cm^−3^), with a design in which P-donors can experience enhanced dielectric confinement effect. **b** Schematic depiction of channel potential with some P-donors having deeper ground state energy level. Such donors will be observed in tunneling transport at higher temperatures. **c** Temperature dependence of *I*_D_-*V*_G_ characteristics (*V*_D_ = 5 mV), showing a SET current peak emerging at the highest temperature of ~100 K [31]

We implement this concept in the design of nanoscale SOI-FETs, in particular in the shape of the channel. We designed stub-shaped-channel SOI-FETs (as shown in Fig. 5a), in which sharp corners of the channel could provide favorable conditions for enhancement of dielectric confinement effect, as illustrated schematically in Fig. 5b for one P-donor. As shown in Fig. 5c, for the smallest such devices, we found that new current peaks emerge at lower *V*_G_ values by increasing temperature. These newly emerging peaks were attributed to single-electron tunneling through P-donors with deeper ground states (see Fig. 5b); tunneling through such deeper-level P-donors could not be observed at lower temperatures because of the low tunneling rates. The final current peak appears at a temperature of about 100 K, which is one of the highest temperatures at which single-electron tunneling via dopant-QDs has been reported so far [31]. From the Arrhenius analysis of the barrier height, we found that the donor giving rise to this final current peak has a barrier height larger than 100 meV (> > 45 meV, ionization energy of P-donors in bulk Si) [31]. This is consistent with an enhancement of the dielectric confinement effect, suggesting a possible pathway toward achieving higher tunneling-operation temperatures for single-dopant transistors [35]. Controlling the design and pattern in such extremely small scales remains, however, significantly challenging and a serious hurdle in front of future development of this approach, but such small structures can be studied in details by first-principle simulations in order to predict useful properties [36].

Alternatively, it may be expected that several P-donors closely located to each other (forming cluster-like configurations) can also lead to a QD with enhanced ionization energy. This cannot be the situation for the present devices, doped with a moderate doping concentration (*N*_D_ ≈ 1 × 10^18^ cm^−3^) since the probability of formation of such multiple-donor clusters is reduced. This case will be discussed in the next section that deals with devices that have highly-doped channels.

#### Transport Via Clusters of Several Dopants

Although single dopants represent a fundamental level of control for electron transport in silicon nanodevices, for many practical applications it can be more useful to introduce a number of strongly-coupled P-donors to form the transport-QD. It was reported that, for silicon transistors with highly-doped channels, some disordered QDs can be formed due to the non-uniform distribution of dopants [37–39]. In our work, we dope our nanoscale-channels with higher doping concentration (*N*_D_ ≈ 5 × 10^18^ − 1 × 10^19^ cm^−3^) in combination with the use of a selective-doping technique to allow more efficient depletion of the heavily-doped channels. We applied this method to fabricate narrow-channel SOI-FETs with selective doping done through a window (slit) having a final width of ~50 nm opened in a SiO_2_ doping mask layer [19]. For these devices, it is critical to control as much as possible the thermal budget after doping, in order to minimize lateral diffusion of dopants within the nominally non-doped barriers. A schematic illustration of the channel of such a device is shown in Fig. 4c.

**Fig. 6:**
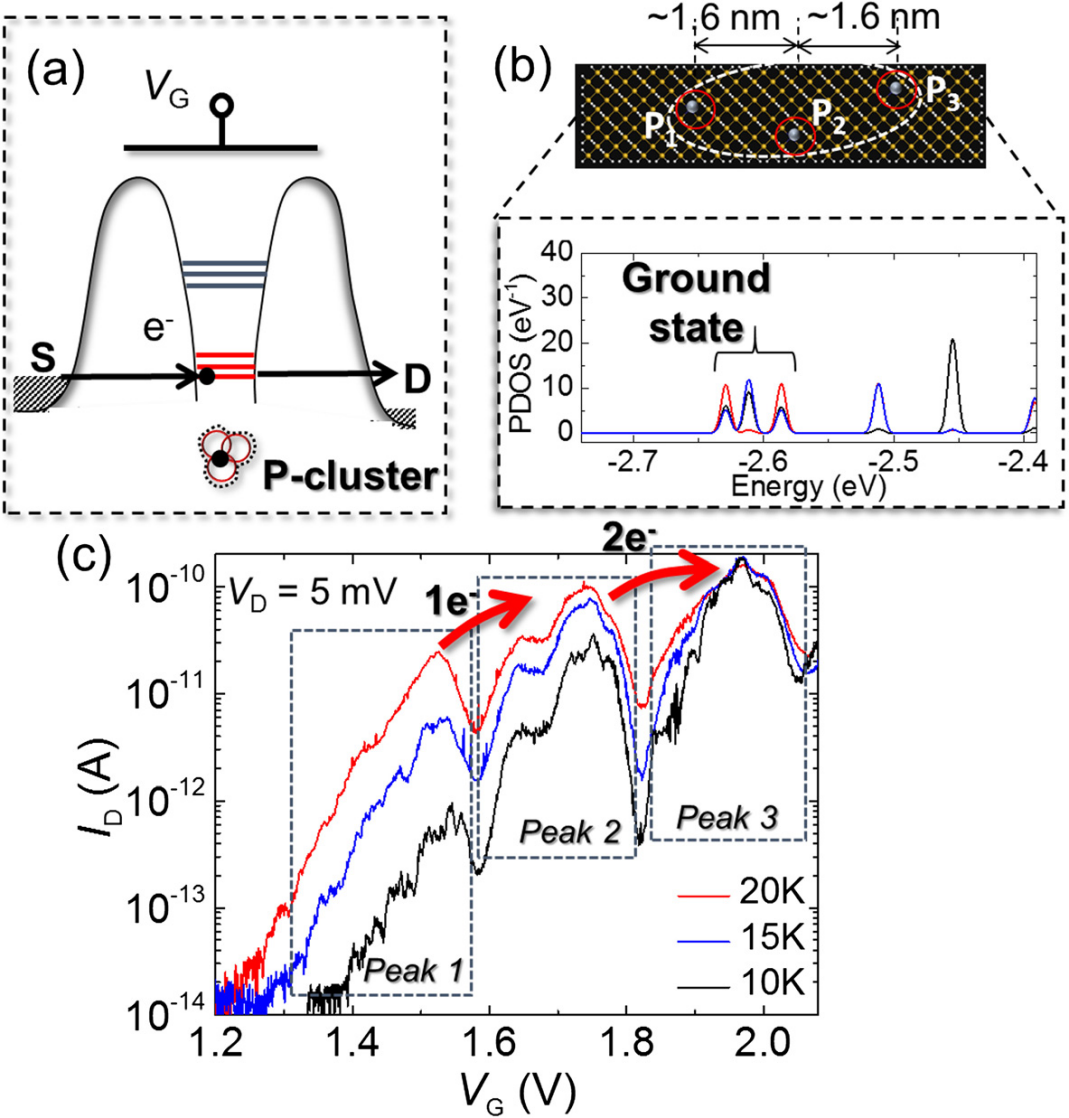
Single-electron tunneling via a cluster of several strongly-coupled donors. **a** Schematic illustration of a QD formed by several strongly-interacting P-donors; the QD exhibits a molecular-like energy spectrum. **b** First-principles calculation of projected density of states (PDOS) spectrum for a 5-nm-long Si nanostructure containing a small number (3) of interacting P-donors. PDOS is plotted by different colors at the location of different P-donors, indicating the splitting of the ground state energy levels. **c** Low-temperature *I*_D_-*V*_G_ characteristics, exhibiting a number of consecutive current peak envelopes (as marked by *dashed boxes*). Each current peak envelope contains steps (inflections) as modulations of the tunneling current due to discrete energy states induced by strongly coupled P-donors [19]

A schematic potential profile induced by several interacting P-donors in such selectively-doped SOI-FETs is shown in Fig. 6a. A relatively complex energy spectrum is expected due to interactions among a number of P-donors located closely to each other. This model is basically supported by first-principles simulations of silicon nanostructures containing a small number of P-donors [19, 36]. The results, such as the example shown in Fig. 6b, suggest that there is a certain correlation between the number of P-donors coupled together and the energy spectrum of the silicon nanostructures. Further evidence has been obtained from the analysis of KPFM measurements correlated with simulations of dopant-induced potential landscapes [25, 26]. Typical *I*_D_-*V*_G_ characteristics for devices of this type are shown in Fig. 6c for a narrow range of low temperatures. Different than the cases for lower-concentration devices, we can observe current peak envelopes, rather than isolated current peaks. These current peak envelopes exhibit a more complex sub-structure, with a number of inflections and sub-peaks incorporated within the envelopes. As described above, these features can be ascribed to tunneling transport through a complex energy spectrum of the transport-QD. Such a complex spectrum is likely induced by the strong interactions among a number of closely placed P-donors.

It is important to note that such molecular-like systems, with a rich energy spectrum and tunability of their properties, can also work as fundamental units for dopant-based devices, but they are significantly different than the case of individual, atomic P-donors. With further optimization of the selective-doping technique and additional understanding of inter-dopant coupling physics [25, 26] at the fundamental level and in extremely scaled-down structures, it can be expected that these systems can offer another pathway toward achieving high-temperature tunneling operation of dopant-based devices.

### Pathways Toward High-Temperature Tunneling Operation of Dopant-Atom Transistors

Based on the discussion presented above, both for low-concentration and high-concentration regimes, we can identify several important factors that may play dominant roles in realizing tunneling operation of dopant-atom transistors at more elevated temperatures. This remains as a target for supporting the feasibility of such fundamental devices for practical applications. In fact, the physics involved in higher-temperature operation of dopant-based devices is practically the same involved in the operation of SETs made with lithographically-defined QDs. Therefore, it is reasonable to treat the efforts to achieve high-temperature operation in parallel for both dopant-atom devices and SETs.

**Fig. 7:**
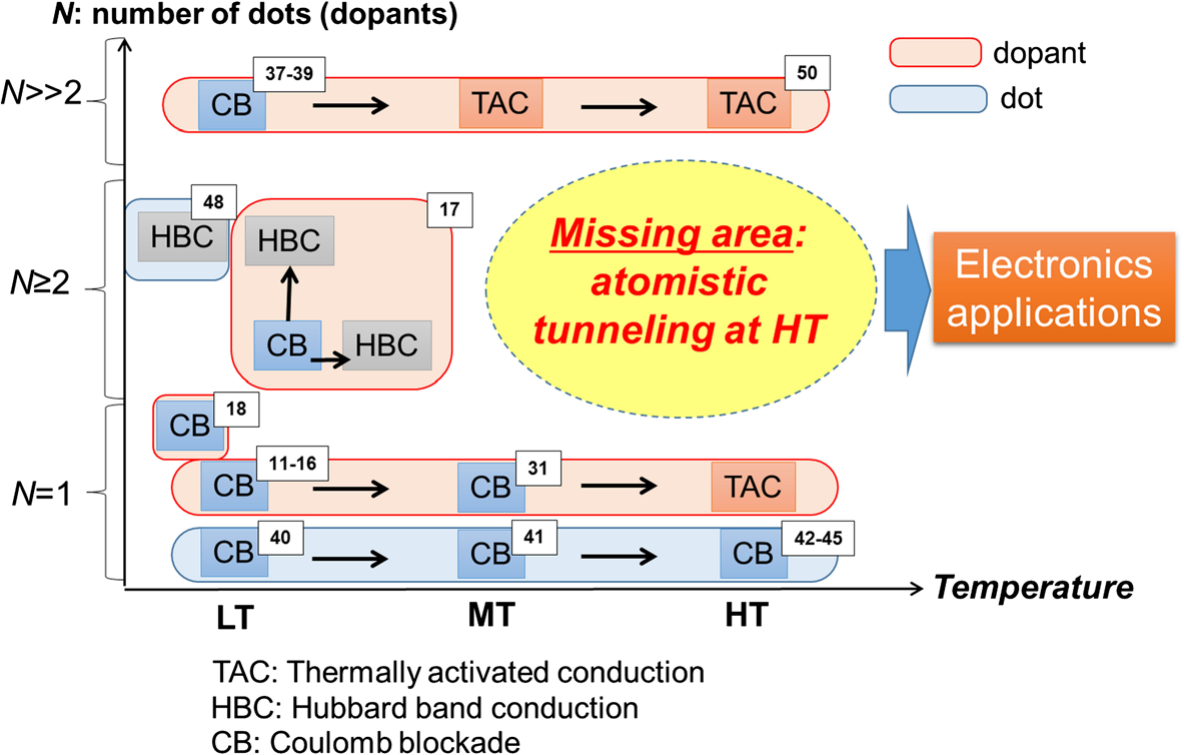
Overview of key factors toward high-temperature tunneling operation. Research directions and critical factors for conduction modes of SETs (with *lithographically defined dots*) and dopant-atom transistors. The diagram is displayed as a function of number of dots or dopants involved in transport (*vertical axis*) and temperature in low, medium, high range, i.e., LT, MT, HT (*horizontal axis*). Relevant references for each conduction mode are also indicated in the diagram

Figure 7 provides an overview of the factors critical for tunneling operation in different temperature ranges (horizontal axis) and as a function of the number of dopants forming the QD or number of QDs involved in transport (vertical axis). Coulomb blockade (CB) is the ideal transport mechanism in which the current is purely given by single-electron tunneling via the dopant-QD (or lithographically defined QD) [11-18, 31, 40-45], without interference due to thermally activated conduction (TAC) component [42]. As temperature is increased, the conduction mechanism changes to TAC and the thermally activated component of the current rapidly masks the CB component. For special cases of a small number of dopants (or QDs) in series, Hubbard band conduction (HBC) [46, 47] may also become a significant current component and it should be treated in conjunction with the CB mechanism. At low temperatures, HBC mechanism has been well studied in previous works [48]. When the number of dopants becomes very large, we can refer to other types of devices such as junctionless transistors [49] and their temperature evolution [50]. Relevant references are indicated in the diagram as well for further details. It should be noted, however, that at present, there is a missing area of experimental results, represented in the diagram as atomistic tunneling at high temperature (HT). This is of critical importance for advanced electronics applications, and it should be directly addressed as a target of future studies, building upon the accumulated knowledge, as illustrated in this diagram. It should not be assumed, however, that this diagram offers an exhaustive overview of all possible factors involved in the tunneling mechanism at high temperatures.

## Conclusions

We provided an overview of several recent results obtained for dopant-atom transistors in which dopants are introduced by thermal-diffusion doping in nanoscale-channels of SOI-FETs. We presented direct observations of dopant-induced potential landscapes measured using a KPFM technique, which show how individual P-donors or “clusters” of closely-placed P-donors modulate the channel potential. Electrical characteristics measured at low temperature reveal that transport occurs in these devices by single-electron tunneling mechanism. The nature of the QD is basically controlled by doping concentration and channel design. Generally, in low-concentration regime, single-electron tunneling via single P-donors is the usual transport mechanism. In the high-concentration regime, tunneling transport occurs through QDs formed by “clusters” of several P-donors located close to each other, inducing a molecular-like energy spectrum. Access to these different regimes of inter-dopant interaction is a first step toward designing and implementing practical applications that utilize systems of one or multiple dopant-atoms. This approach can provide insights into functionalities arising from atomic and, respectively, molecular systems built into the conventional Si-based electronics platform.
